# An Atypical Presentation of Burkitt Lymphoma Presenting As Large Intra-abdominal Masses Compressing Multiple Organs With Peri-Pericardial Involvement in an HIV Patient: A Case Report and Literature Review

**DOI:** 10.7759/cureus.54088

**Published:** 2024-02-12

**Authors:** Derek Ugwendum, Annmarie Fernando, Divine Besong Arrey Agbor, Nkafu Bechem Ndemazie, Sabastain F Forsah, Ikpechukwu J Okorie, Kevin Villanueva, Angela Grigos, Jay Nfonoyim

**Affiliations:** 1 Internal Medicine, Richmond University Medical Center, Staten Island, USA; 2 Radiology, Richmond University Medical Center, Staten Island, USA; 3 Pulmonary and Critical Care, Richmond University Medical Center, Staten Island, USA

**Keywords:** epstein-barr virus, human immunodeficiency virus, antiretroviral therapy, chemotherapy, burkitt lymphoma

## Abstract

Many types of malignancies have been associated with immunodeficiency states, especially patients who are HIV positive. Burkitt lymphoma (BL) is one of those malignancies associated with HIV and it presents in three varieties. The endemic form is primarily seen in children, and it is associated with the Epstein-Barr virus (EBV). In this form, patients with Burkitt’s present with a large jaw mass. The second variety is seen in older adults. These patients usually present with abdominal and pelvic masses. This subtype is more prominent in the United States. The third variety of BL is seen in patients who are HIV positive. In this case report, we present an atypical presentation of BL secondary to undiagnosed HIV/AIDS with a very large tumor burden causing compressive symptoms. This case will further guide healthcare professionals in diagnosing BL, which presents uniquely in high-risk populations. This report will also serve as a review of the diagnosis and treatment options of BL.

## Introduction

Burkitt lymphoma (BL) is one of the malignancies that is associated with the human immunodeficiency virus (HIV), which currently remains a global health concern. HIV can be transmitted from an infected person to an uninfected person through the exchange of body fluids such as breast milk, vaginal secretions, semen, and blood [1,2]. There are conditions and behaviors that increase the risk of contracting HIV; one of which is having anal and vaginal sex without the use of condoms [3]. In this case presentation, we discuss a patient who engages in sexual intercourse with other men, who was unaware that he was HIV positive, presenting with multiple large abdominal masses compressing abdominal organs. The biopsy result of the masses was positive for BL. This is an uncommon presentation of BL with a patient presenting with an oral BL mass and compressive features both on laboratory and clinical presentations secondary to the large abdominal masses (BL).

## Case presentation

A 47-year-old male with no past medical history, who has sex with men and with about 20 pack years of smoking history, presented to the emergency department with a two-week history of intractable vomiting and abdominal pain. He endorsed non-bloody and non-bilious vomitus, which was unprovoked in nature and worsened postprandially. He also complained of an unquantifiable weight loss, fatigue, shortness of breath, and constipation.

On examination, he had a temperature of 98.1°F, heart rate of 88 bpm, blood pressure of 112/75 mmHg, and respiratory rate of 16 bpm. Blood oxygen saturation was 98% on room air. Physical examination was remarkable for jaundice, a non-tender dental mass involving the left molars, poor dental hygiene, multiple palpable abdominal masses, and a palpable left testicular mass. Initial laboratory studies demonstrated hyponatremia (128 mEq/L), hypochloremia (86 mmol/L), increased renal function tests (blood urea nitrogen: 28 mmol/L, creatinine: 1.45 mg/dL), elevated lactic acid (4.4 mmol/L), total bilirubin (6.3 mg/dL), direct bilirubin (4.5 mg/dL), aspartate aminotransferase (AST) (80 U/L), alanine transaminase (ALT) (86 U/L), alkaline phosphatase (ALP) (270 U/L), lipase (318 U/L), and lactate dehydrogenase (387 U/L). 

Both HIV1 and 2 DNA genotypes were detected, deeming the patient to be HIV positive with a viral load of 23000 copies/mL. Further results indicated the percentage of CD3/CD4 and CD3/CD8 were 23 and 55, respectively. Hepatitis viral panel (hepatitis B and C) was negative.

**Figure 1:**
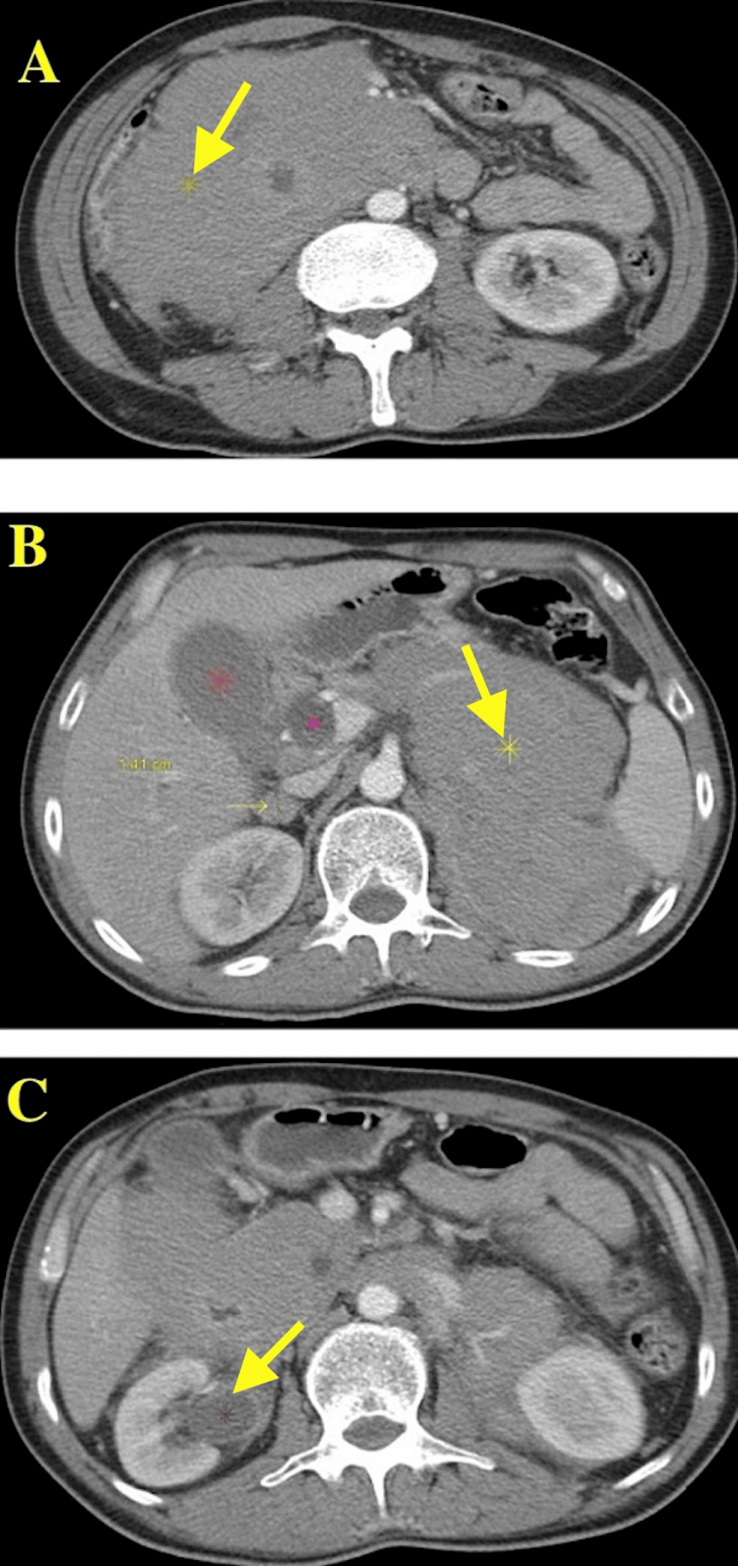
CT abdomen and pelvis. Panel A shows a large right upper quadrant mass. Panel B shows a left upper quadrant mass with gallbladder distension, minimal wall thickening, and some pericholecystic fluid. Panel C: right renal nodule measuring 1.4 cm with right-sided hydroureter most likely due to extrinsic compression from the mass. CT, computed tomography

Computed tomography (CT) of the abdomen was requested revealing a large upper quadrant abdominal mass on the right side in the subhepatic space extending toward and by the pancreatic head and porta hepatis. Another large upper quadrant mass in the suprarenal space was found adjacent and medial to the spleen. This mass extended down to the renal hilum. The gallbladder was distended with mild wall thickening and pericholecystic fluid. There was also dilatation of the pancreatic duct and the common bile duct due to the external compression by the mass. The mass was presumed to be arising from the pancreatic head. There were also additional masses in the pancreatic body and tail. A nodule within the right adrenal gland was also noted. The right kidney demonstrated hydronephrosis (Figure 1).

An echocardiogram was done, which showed normal global left ventricular systolic function with a left ventricular ejection fraction of about 60-65%. There was a trivial posterior echo-free space complicated with a small pericardial effusion and subepicardial fat.

The patient was admitted to further investigate these findings. The hematology/oncology, nephrology, urology, infectious disease, gastroenterology, ENT, surgery, and palliative care teams were consulted.

Further testing included an ultrasound of the left testicle, which indicated a hypoechoic structure in the inferior lateral aspect of the scrotum. This structure was independent of the testicle.

**Figure 2:**
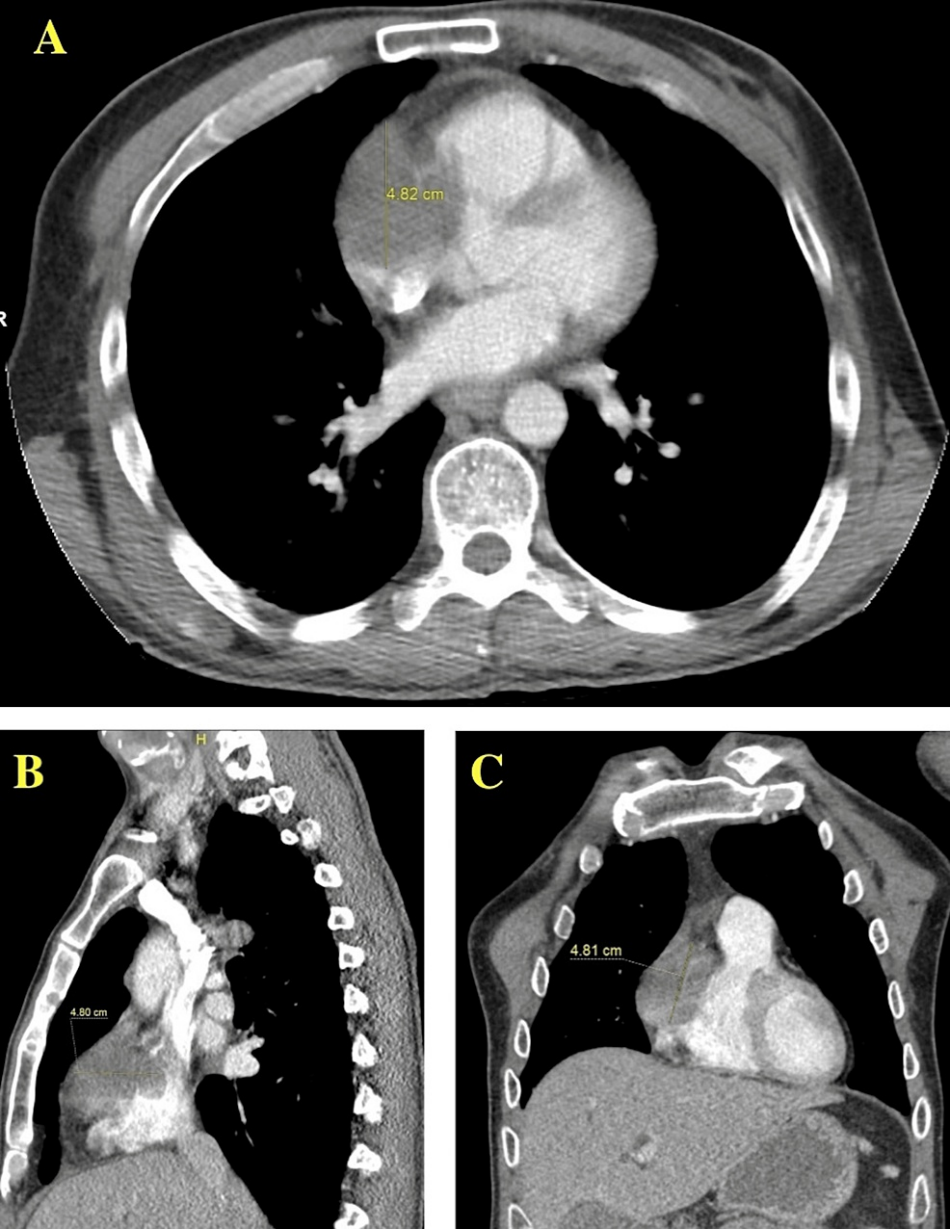
Chest CT with panels A, B, and C showing a 4.8 cm mediastinal mass. CT, computed tomography

Additionally, CT of the chest indicated a right pericardial mass and a pleural-based right middle lobe nodule as shown below in Figure 2.

**Figure 3:**
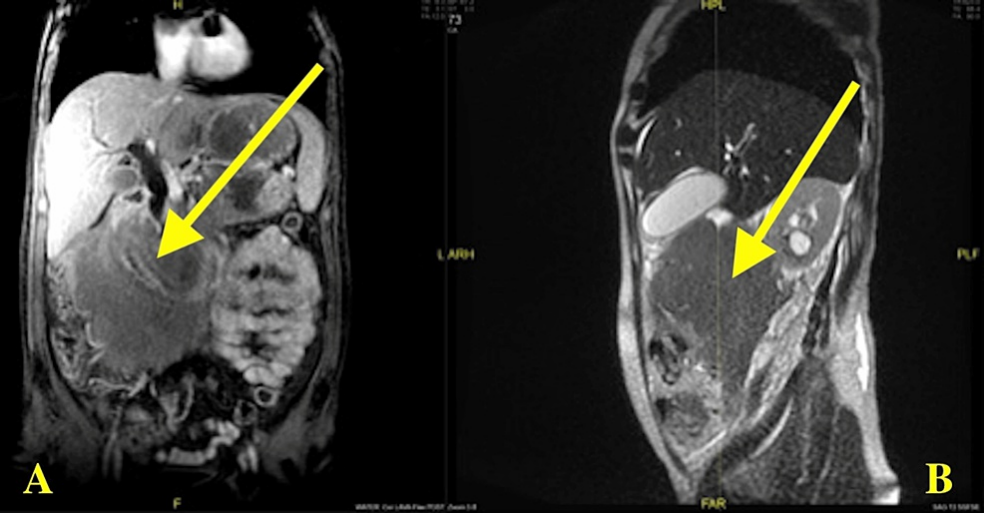
MRI abdomen and pelvis with panels A and B showing two large peritoneal masses displacing the pancreas anteriorly, spleen laterally, and the right kidneys inferiorly. MRI, magnetic resonance imaging

Magnetic resonance imaging (MRI) of the abdomen was remarkable for two large retroperitoneal masses. The left mass measured about 14.9x13.8x10.2 cm and was displacing the pancreas anteriorly, spleen laterally, and kidney inferiorly. The mass was also seen to be extending to the aorta and was partly surrounding the celiac trunk and superior mesenteric artery. This mass was noted to be connected to another large mass measuring 16.2x17x13.8 cm. This second mass was seen to surround the second and third part of the duodenum and was inseparable from the pancreatic head. This resulted in moderate pancreatic duct dilatation and severe biliary duct dilatation. This mass extended to the right hilum, surrounded and severely narrowed the right renal pelvis and proximal ureter, resulting in moderate hydronephrosis. The left retroperitoneal mass also extended to the left renal hilum and surrounded the left renal artery and segmental arteries. A nodule was noted in the lateral limb of the right adrenal gland (Figure 3).

CT of the neck indicated soft tissue fullness in the left palatine tonsillar region, which was concerning for malignancy. The left jugulodigastric lymph node was also noted to be measuring 2x1.5x3.2 cm.

**Figure 4:**
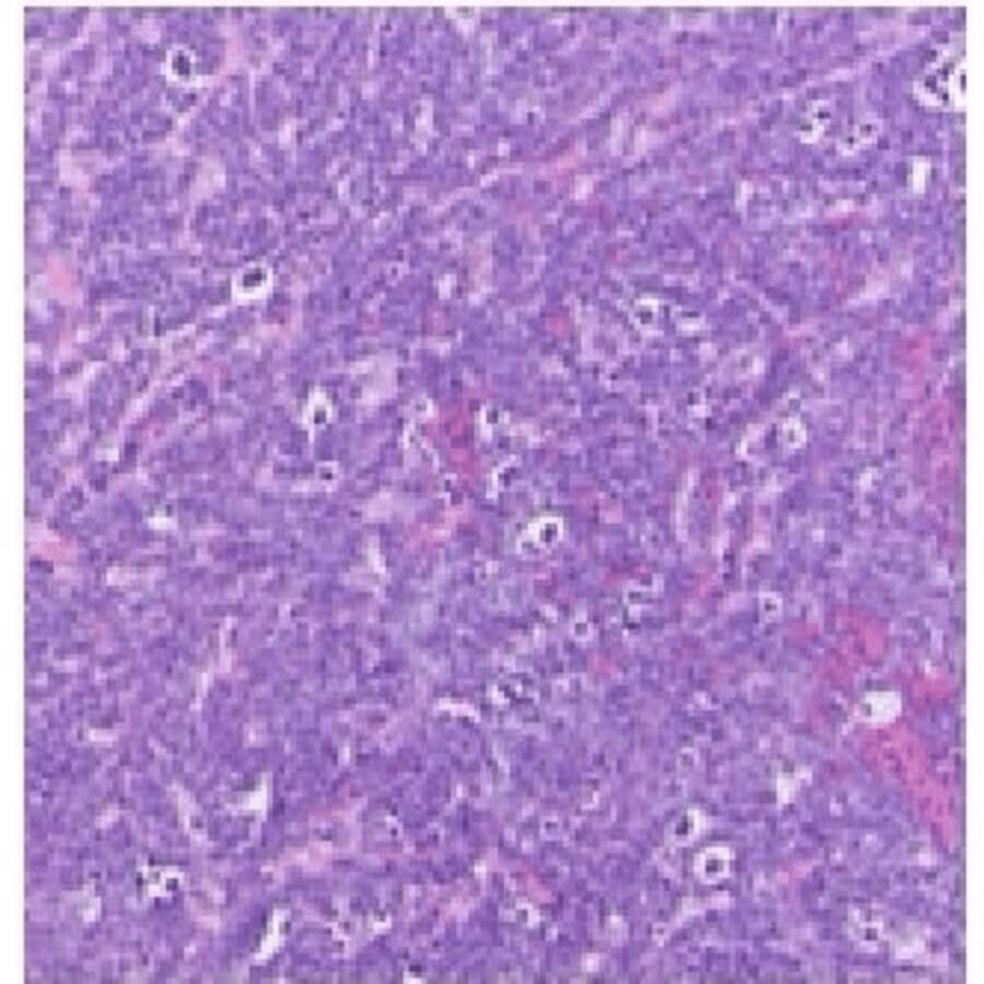
Pathology report showing BL. BL, Burkitt lymphoma

CT-guided biopsy for the intra-abdominal mass was conducted. The surgical pathology report (Figure 4) concluded BL with translocation of t(8;14). It showed translocation of the Myc gene and IgH gene. It was positive for CD20, CD10, BCL-2, BCL-6, CD3, CD5, Ki-67 (positive, 100% tumor cell), Kappa, and negative for Lambda, Cyc-D1, and CD30.

The pathology report of the right gingival mass was also indicative of BL. Cerebrospinal fluid analysis and MRI of the brain were negative for the involvement of BL. Bone marrow biopsy also was negative for the involvement of lymphoma.

He was started on Biktarvy® (bictegravir 50 mg, emtricitabine 200 mg, and tenofovir 25 mg) one tablet per day and was placed on prophylaxis with trimethoprim-sulfamethoxazole 480 mg daily. The pain was well controlled with morphine. He received methotrexate 12 mg intrathecally and a chemo port was placed. A short-duration dose-adjusted combination chemotherapy EPOCH-R (etoposide, doxorubicin, vincristine, rituximab, prednisone) and cyclophosphamide were initiated. The patient was administered rasburicase 14 mg once with normal saline at a rate of 100 mL/hour to prevent tumor lysis syndrome when chemotherapy was initiated. Tumor lysis syndrome was monitored, and he was continued on allopurinol 300 mg daily.

Upon completion of his first round of chemotherapy, the patient's tumor responded well to chemotherapy. Both his clinical symptoms and laboratory results (as seen in Table 1 below) improved. He was discharged with allopurinol, antiretroviral therapy (highly active antiretroviral therapy (HAART)), and trimethoprim-sulfamethoxazole with the plan of completing his chemotherapy in an outpatient setting. He was advised to follow up twice a week for blood work.

**Table 1:** Laboratory results during hospitalization, pre- and post-chemotherapy treatment period. AST, aspartate aminotransferase; ALT, alanine transaminase; ALP, alkaline phosphatase; BUN, blood urea nitrogen; Cr, creatinine; RF, reference range

Days of hospitalization	RF	D1 (day of admission)	D2	D3	D4	D5	D6	D7	D8 (day of chemotherapy)	D9	D10	D11	D12
AST	<34 U/L	80	56	106	109	136	117	99	147	259	246	83	18
ALT	10-49 U/L	86	49	62	81	88	88	75	75	107	125	45	34
ALP	46-116 U/L	270	166	276	334	373	343	292	278	304	248	211	111
BUN	7-18 mg/dL	24	28	30	32	30	28	27	28	24	22	15	17
Cr	0.70-1.30 mg/dL	1.45	1.39	1.30	1.46	1.28	1.33	1.30	1.33	1.20	1.16	0.89	0.78

## Discussion

BL is an aggressive high-grade B-cell lymphoma that was first described by Denis Burkitt [4]. There are three subtypes according to the World Health Organization, which include endemic type, sporadic type, and immunodeficiency-associated disease type [5]. Endemic BL is the type that is commonly seen in equatorial Africa and most prevalent malignancy among children in sub-Saharan Africa with a 2:1 male-to-female ratio. It is the type that is associated with the Epstein-Barr virus (EBV). Patients with this type usually present with an enlarging mass of the jaws [6,7]. The sporadic form occurs anywhere in the world without a particular geographic location. In the USA and Western Europe, it accounts for about 1% to 2% of cases of lymphoma in the adult population. EBV is less associated with the sporadic form compared to the endemic type [8]. This form involves extra-nodal locations especially the central nervous system, bone marrow, and gastrointestinal tract [8,9]. The third type is that which is associated with immunodeficiency with HIV being the most common etiology as seen in our case. In the United States, it accounts for about 20% of cases of BL [7]. It is seen in patients with higher CD4 counts, and its prevalence has not changed even with HIV patients on highly active antiretroviral therapy [10]. This type presents with nodal involvement, CNS, liver, lungs, and bone marrow [11,12].

When BL is suspected, the diagnosis should be initiated promptly because of the rapidly progressive nature of the disease irrespective of the form. Baseline workup includes laboratory tests, imaging, HIV screening, hepatitis B screening, lumbar puncture with flow cytometry, and cytology of the cerebrospinal fluid for CNS involvement. Biopsy for histology and immunohistochemistry of the tumor mass helps with definitive diagnosis. On histology, BL is characterized by lymphoid cells, which are neoplastic with a small proportion of amphophilic cytoplasm, dispersed chromatin, round nuclei, and many small nucleoli. The tumor cells are mixed with tingible body macrophages (these macrophages are phagocytosing apoptotic debris), thus giving a "starry sky" appearance. On immunochemistry, most often, this tumor has malignant cells that are immunoreactive to immunoglobulin M and are positive for CD10, CD20, CD22, CD79a, BCL6, HLA-DR, and CD45 and are not immunoreactive to CD5, BCL2, CD23, and TdT [13-16].

On nuclear staining, these tumor cells are positive for the c-Myc gene. Worth noting, in all BL cases, the cMyc gene translocates with either the IgH gene in about 80% of cases or the kappa/lambda light chain gene in approximately 20% of cases [17]. Our patient was confirmed on pathology to have BL, and as part of the baseline work-up, HIV screening was done that resulted positive. His pathology report showed translocation of the Myc gene and IgH gene. His biopsy was positive for CD20, CD10, BCL-2, BCL-6, CD3, CD5, Ki-67 (a measure of growth fraction), and kappa and negative for lambda, Cyc-D1, and CD30.

Typically, BL treatment consists of high-dose chemotherapy that is intermittently administered. Being a rapidly growing tumor, it is highly sensitive to chemotherapy and is one of the cancers in the past for which cures were achieved with only chemotherapy [18,19]. Commonly used chemotherapy includes cyclophosphamide, vincristine, doxorubicin, and methotrexate. Rituximab, a monoclonal CD20 antibody, has shown significant promise when combined with the typical chemotherapy regimen [20]. Other chemotherapeutic regimens that have been used include the R-EPOCH regimen, which constitutes a dose-adjusted rituximab, etoposide, prednisone, vincristine, cyclophosphamide, and doxorubicin [21,22]. The CODOX-M/IVAC regimen comprises cyclophosphamide, doxorubicin, vincristine, methotrexate, ifosfamide, cytarabine, and etoposide [23]. Surgical interventions such as tumor debulking can be beneficial as an adjunct treatment. This is employed when the anatomical location of the tumor is permissive. This method of treatment has not been well studied in head and neck BL presentations [24,25]. In patients with poor prognosis, palliation therapy with locoregional radiation helps to control local symptoms [26].

Our patient was on EPOCH-R regimen (etoposide, doxorubicin, vincristine, rituximab, prednisone) and cyclophosphamide. After the course of his first regimen, his tumor responded well by a rapid shrinkage of his abdominal tumor. His kidney functions and obstructive liver function test improved significantly. His obstructive gastrointestinal symptoms of nausea, vomiting, and abdominal discomfort significantly improved. He was also on HAART, Biktarvy® one tablet daily, and trimethoprim-sulfamethoxazole for prophylaxis against opportunistic infections. Our patient was on allopurinol because BL has a high rate of cellular proliferation and cellular turnover thus tumor lysis syndrome which is characterized by hyperuricemia, hyperkalemia, hyperphosphatemia, and hypocalcemia [27].

## Conclusions

HIV infection is the most common cause of BL associated with immunodeficiency as seen in our case. Our patient presented with an atypical presentation of BL. He had a very large intra-abdominal mass with multiorgan system compression that featured both on clinical presentation and on laboratory findings with obstructive patterns in terms of liver function and pancreatic enzymes. This helps to highlight that patients with BL can have such presentations. Thus, it should be considered as a differential when a patient presents with large intra-abdominal tumors with compression features.
